# Pseudo-Scientific Credulity

**Published:** 1899-03

**Authors:** 


### PSEUDO-SCIENTIFIC CREDULITY.

It is a long time since New Englanders have burned a witch. The advance of civilization has been accompanied by a general enlightenment that has shaken off from the tree of knowledge a large portion of its imperfect adventitious fruit. We can now only ridicule those diabolical concoctions which marked the therapeutics of the Middle Ages. "Witches, mummy, maw and gulf of the ravin'd salt-sea shark," and the other delicacies which made "thick and slab" the gruel compounded by Macbeth's elderly female friends and counselors; poultices of bovine excrement; amulets and charms of claws; even innocent necklaces of amber—all these relics of an age of superstition and crass credulity have lost their hold upon the professional and popular mind.

In modern times, however, with the development of the laws and truth of natural science—perhaps in part as a result of the almost miraculous inventions and discoveries of a scientific character that have attended this development—the remnant of that medieval superstition and credulity has at different periods in these later days attached itself with more or less tenacity to chimerical projects of a pseudo-scientific sort. Recent years afford no more striking proof that these instincts still thrive in our midst, and no more brilliant illustration of the extent to which a cunning shrewdness may prey upon them, than is afforded by the checkered career of the late Keeley, of "motor" fame. Exploited, petted, and fostered throughout his career by men of prominence in intellectual and commercial circles, he seems to have held them all fascinated, solely by the appeal his preposterous theories made to their credulity. It is reported that when an examination was made, after his demise, of his complicated "motor" and its intricate accessories, there was discovered a large steel sphere, capable of storing compressed air under very high pressure, placed in a hidden but convenient location, which supplied through a very small tube (passing for a conducting wire) the power to influence the "motor." There seems now to have been no basis whatever in his alleged discoveries of a new motive power or "influence." And yet for nearly a quarter of a century this man had been able to interest in his scheme not only intelligent people of well-known ability, but also to procure the aid of no small amount of capital. This seems to have been an instance in which the sort of credulity that has for its basis a smattering knowledge of mechanics drew a glowing veil of false enthusiasm over the usually calculating and unemotional eye of capital.

Apparently the field of therapeutics affords pasture as verdant, rich, and sustaining as that of mechanics. Not only is that variety of quackery which exists beyond the pale of legitimate medical practice fed and supported by popular credulity, but there is grave reason for the suspicion that not a little of this medieval quality of mind still infests the regular profession. The tons of alleged literature annually sent to medical men, lauding various proprietary remedies, abounding in lurid testimonials and reports of marvelous cures, would cease to burden the postal department and choke the engulfing maw of the physician's waste-basket were there not some response to these appeals, that renders the enormous circulation of such literature pecuniarily profitable.

There are some undesirable qualities in the human mind that seem to resist enlightenment with great pertinacity, and of all these qualities, that form of credulity which has a sort of pseudo-scientific basis seems most to thrive in our modern life. "The earth hath bubbles as the water has, and these are of them."—*Medical Age.*

